# Dense sampling for mapping pituitary growth dynamics before, during, and after pregnancy

**DOI:** 10.1111/jne.70141

**Published:** 2026-02-05

**Authors:** Giorgia Picci, Risha Arora, Hannah Grotzinger, Kaya Jordan, Laura Pritschet, Elizabeth R. Chrastil, Emily G. Jacobs, Jerod M. Rasmussen

**Affiliations:** ^1^ Institute for Human Neuroscience, Boys Town National Research Hospital Boys Town Nebraska USA; ^2^ Center for Pediatric Brain Health, Boys Town National Research Hospital Boys Town Nebraska USA; ^3^ Department of Pharmacology and Neuroscience Creighton University Omaha Nebraska USA; ^4^ Department of Pediatrics University of California Irvine California USA; ^5^ Department of Psychological & Brain Sciences University of California Santa Barbara California USA; ^6^ Ann S. Bowers Women's Brain Health Initiative Santa Barbara California USA; ^7^ Department of Psychiatry University of Pennsylvania Philadelphia Pennsylvania USA; ^8^ Department of Neurobiology and Behavior University of California Irvine California USA; ^9^ Department of Biomedical Engineering University of California Irvine California USA; ^10^ Department of Anatomy and Neurobiology University of California Irvine California USA

**Keywords:** dense phenotyping, HPA axis, MRI, pituitary, pregnancy

## Abstract

Pregnancy represents a period of profound endocrine activity and neural reorganization. While recent evidence highlights pituitary volume as a biomarker of endocrine dynamics during pregnancy, its precise trajectory (timing and relative magnitude of effect) across human pregnancy remains undescribed. Three healthy women (59 total observations) underwent T1‐weighted MRI before conception (5 baseline observations), during pregnancy (38 total observations, spanning gestational weeks 1–36), and up to 1 year postpartum (16 total observations). Anterior and posterior pituitary lobes were manually delineated at every visit. A longitudinal pipeline co‐registered each scan to all other intra‐subject scans, propagated their labels, and generated majority‐vote ensembles for objective and regularized volume estimates. Person‐centered z‐scores were computed, and generalized additive mixed models (GAMMs) with random intercepts estimated nonlinear volume trajectories. The anterior lobe followed a nonlinear trajectory, with gestational age explaining 73% of adjusted variance in anterior‐pituitary volume (edf = 7.59, F = 20.2, p_bonf_ < 10^−10^). Specifically, volume exhibited a modest first trimester decrease (local fit minima: −0.9 SD at 10.6 weeks), followed by a steep rise into the 3rd trimester (local fit maxima: +1.8 SD at 34.1 weeks, or ~ 17.5% increase from 1st trimester minima, by volume), before returning to baseline near 3 months postpartum. Sensitivity analyses restricted to linear regression during early (−5 to 12 weeks) and late (12 to 40 weeks) windows replicated the observed non‐linear decreasing/increasing slopes (β_early_ = −0.09 SD/week, p_early_ = 0.036; β_late_ = 0.14 SD/week, p_late_ < 10^−10^). In contrast, no significant volumetric changes in the posterior lobe were detected across the observation period (p_non‐linear_ = 0.79). In one of the first studies of its kind to leverage a dense sampling approach in multiple pregnant women, non‐linear analyses revealed rapid, reversible anterior pituitary hypertrophy across human pregnancy consistent with lactotrope expansion and heightened endocrine load.

## 1 INTRODUCTION

Pregnancy is a period of profound endocrine activity and neural remodeling, including well‐documented adaptations in the functional and structural properties of the maternal brain.^1^, ^2^, ^3^ Such adaptations underlie parenting behaviors in animal models and are believed to be largely mediated through pituitary activation.^4^ While the hormones released along this axis support healthy growth and development, their dysregulation is linked to a myriad of affective disorders, immune and metabolic complications, as well as delayed recovery postpartum.^5^, ^6^

MRI‐based estimates of pituitary volume have recently been used to define normative trajectories across the lifespan,^7^ serve as biomarkers of transdiagnostic affective symptoms in youth,^8^ and characterize neuroendocrine conditions such as precocious puberty^9^ and Prader‐Willi syndrome.^10^ In the latter, altered T1 signal intensity in the posterior pituitary is believed to reflect disruptions in neuropeptide systems such as oxytocin and vasopressin via lipid‐rich neurosecretory granules. Collectively, these findings support the utility of MRI‐based pituitary measures in clinical and developmental contexts.

MRI‐based pituitary measures support the premise of intense activation during pregnancy, although estimates of the magnitude of volumetric change vary widely. In fact, early cross‐sectional studies using low‐resolution MRI and simple height, width, and length measures suggested that the pituitary nearly doubles in size from the first to third trimester.^11^, ^12^ Such dramatic changes are hypothesized to reflect lactotroph hyperplasia (i.e., pituitary growth related to prolactin production and release) and hypertrophy. Importantly, these changes complicate diagnosis and management of pituitary disorders during pregnancy, further underscoring the need to understand normative peripartum pituitary physiology.^13^ More recently, using a more modern MRI acquisition and what may be the only prospective pre‐pregnancy longitudinal measures of pituitary volume to date, pregnancy induced increases in pituitary volume were observed to be far more modest (~7% increase).^14^ However, no scans we acquired *during* pregnancy, and estimates of the effect of pregnancy relied on measures acquired more than 2 months post‐partum on average. Moreover, studies have overwhelmingly relied on estimates of whole pituitary volume, without focused segmentation of the anterior pituitary, despite its central role in regulating the majority of hormones undergoing dramatic fluctuations during and after pregnancy (e.g., progesterone, estrogen). However, in perhaps the most robust estimate of effect sizes to date, one cross‐sectional study of 68 pregnant women reported an average increase of ~16% in anterior pituitary volume compared to post‐partum and control individuals.^15^ Notably, gestational age at MRI was not analytically considered, thereby limiting inferences about within‐subject dynamics that are expected to markedly shift across pregnancy. Taken together, the extant literature provides initial, largely cross‐sectional, evidence for pituitary gland enlargement during pregnancy. Despite growing interest, precise pituitary volume trajectories across pregnancy remain not yet fully described and would benefit from repeated intraindividual measurements spanning the preconception period, each trimester, and the postpartum phase.

The present study uses a dense sampling approach to address current gaps in the literature by more fully characterizing the rapid dynamics in pituitary volume captured before, during, and after pregnancy. We combine well‐validated manual segmentation protocols with advanced longitudinal image‐based co‐registration methods to provide regularized high spatial and temporal resolution estimates of anterior and posterior pituitary volume in three women across a total of 59 visits. Dense sampling designs offer a powerful framework for capturing intraindividual change processes, such as those occurring during pregnancy,^1^ that would be difficult to otherwise detect using conventional neuroimaging approaches with widely spaced timepoints.^16^ Finally, person‐centered generalized additive mixed models (GAMMs) with random intercepts were used to characterize the within‐person non‐linear response to pregnancy. Collectively, this powerful sampling approach provides a unique window into pituitary remodeling and lays a methodological foundation for future studies linking neuroendocrine dynamics to peripartum outcomes.

## 2 MATERIALS AND METHODS

### 2.1 Sample description and data collection

The participants in this study were healthy primiparous women (ages 26, 34, and 38) enrolled prior to pregnancy (2 in vitro fertilization, 1 spontaneous conception). Participants were without major obstetric complications, delivered near full term (37 weeks 4 days, 39 weeks 1 day, 39 weeks gestation; 1 cesarean section, 2 vaginal deliveries) and nursed throughout postpartum imaging sessions. All participants gave written informed consent and the study was approved by the University of California Irvine Human Subjects Committee.

T1‐weighted MRI image acquisition (MPRAGE, TR/TE/TI = 2500/2.31/934 ms, FA = 7 deg., 0.8 mm isotropic resolution) was conducted on Siemens 3T Prisma scanners at the University of California Irvine and the University of California Santa Barbara. Imaging sessions (59 in total across 3 participants) occurred before conception (5 observations, 7.4 weeks prior [range: 0.5–18]), during pregnancy (38 observations ranging from 1 to 36 weeks gestation), and up to roughly 1 year postpartum (16 observations, 16.7 ± 13.0 weeks post [range: 4–55]) (See Figure S1 for timing and frequency of observations by participant). For a more detailed overview of the imaging and longitudinal design, we refer the reader to Pritschet et al., which describes whole‐brain morphometric changes across pregnancy in one of these participants^2^; here, we extend this work by focusing specifically on pituitary structure and signal characteristics.

### 2.2 Data preprocessing

#### 2.2.1 Manual segmentations

Anterior and posterior pituitary lobes were manually traced using itk‐SNAP (v3.8, itksnap.org) using an existing validated protocol and expert input (GP).^8^ Specifically, the coronal view was used to define borders based on the diaphragm sellae and lateral ventricles superiorly, sphenoid sinus inferiorly, and internal carotid arteries bilaterally. The sagittal view was used to identify the boundary between the anterior and posterior lobes of the pituitary based on marked differences in signal intensity. That is, the posterior portion of the pituitary gland typically appears hyperintense relative to the anterior pituitary due to its high content of neurosecretory granules and associated phospholipids.^17^, ^18^

#### 2.2.2 Semi‐automatic segmentation

**FIGURE 1:**
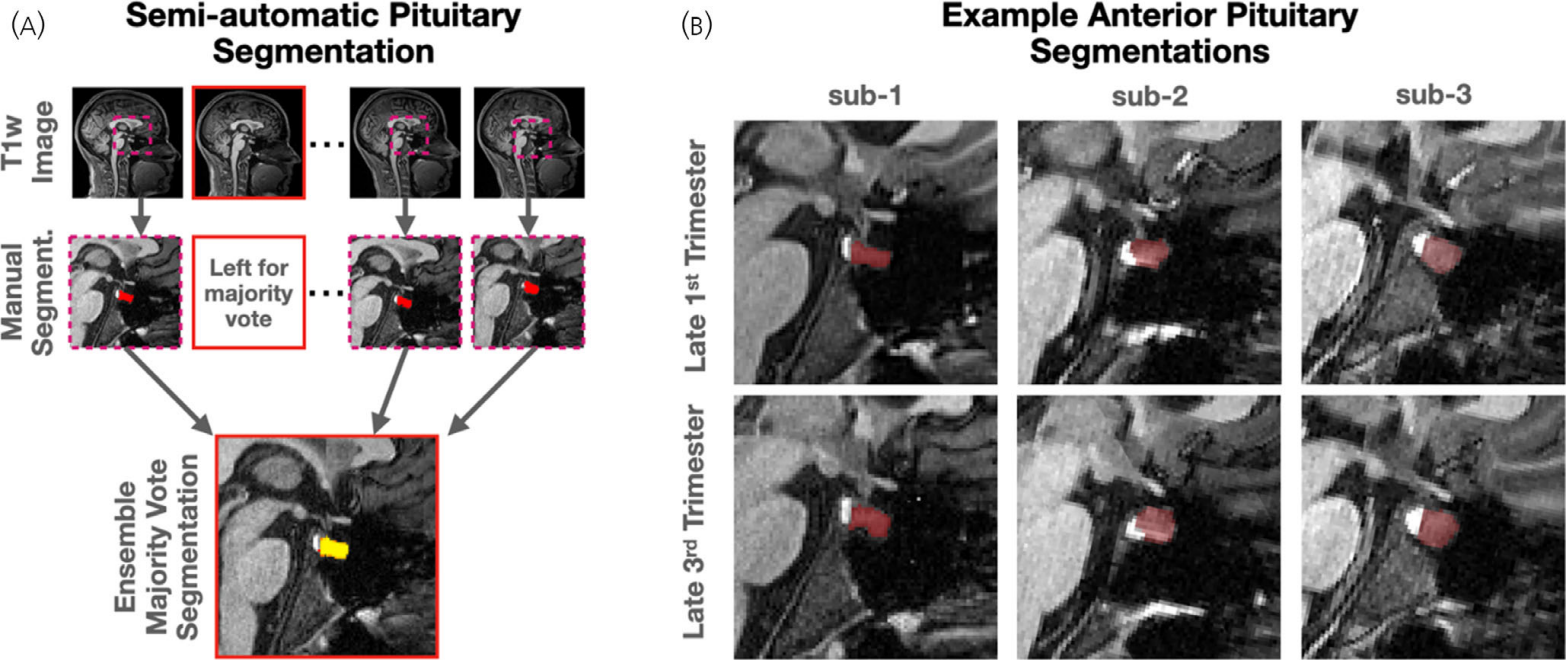
Pituitary segmentation across pregnancy. (A) A majority votes algorithm based on co‐registered within‐subject expert ratings was used as an objective regularized definition of pituitary. (B) Example anterior pituitary segmentations from each subject in early and late pregnancy are shown.

A novel pipeline for longitudinal pituitary segmentation was designed to help reduce noise in border definition by leveraging shared information across imaging sessions. Specifically, within each subject, all other sessions were co‐registered to each individual session using non‐linear symmetric deformation and a majority votes algorithm (Figure 1) was used to define pituitary borders.^19^ This process balances expert delineation of pituitary borders with signal averaging and objective intensity‐based segmentation. Median anterior and posterior T1‐weighted signal intensity was extracted, and relative signal intensity differences were characterized using a two‐sample t‐score. Finally, volume and signal intensity measures were person‐centered (z‐scored) to account for individual differences in absolute pituitary size and image intensity scaling, thereby isolating within‐subject trajectories and ensuring that observed effects reflect biologically meaningful change rather than between‐subject or scanner‐specific variability. Raw measures are reported as Supporting Information.

### 2.3 Data Postprocessing

Generalized Additive Mixed Models (GAMMs) were used to model pituitary trajectories in the context of pregnancy (R v4.3.3; mgcv package). GAMMs natively allow for a repeated measures design and non‐linear fit using heterogenous sampling. Smooth terms (k = 10, default) were applied to gestational age to model non‐linear trajectories, with random intercepts specified for each subject to account for individual baselines. Models estimated within‐subject autocorrelation (⍴) using a continuous first‐order autoregressive correlation [CAR(1)] structure (*corCAR*), which accounts for residual dependence that decays exponentially with increasing time lag between observations. Model diagnostics and first derivative estimates were used to identify periods of significant change in the model fits. Additional post‐hoc analyses using linear mixed‐effects models within defined early (−5 to 12 gestational weeks) and late (12 to 40 gestational weeks) windows were used to characterize linear segments of the trajectory. This approach allowed for more interpretable slope estimates and formal tests of significance. Finally, while we report model fits using variance explained (R^2^), we acknowledge that this is an optimistic measure unlikely to generalize. To address this, Leave‐One‐Out (LOO) predictive performance was assessed via leave‐one‐subject‐out cross‐validation. In each fold, we fit the GAMMs to two subjects and predicted the held‐out subject using the fixed‐effects to report predictive out‐of‐sample variance.

## 3 RESULTS

### 3.1 Manual and semi‐automatic segmentations

All 59 T1‐weighted images available for segmentation were qualitatively observed to be sufficient for high quality manual pituitary segmentation. Raw pituitary volumes were consistent with those observed in prior studies (anterior pituitary volume = 525 ± 29/799 ± 68/799 ± 41 mm^3^; posterior pituitary volume = 92 ± 6/100 ± 8/103 ± 4 mm^3^ on average for each participant, Figure 1). There were no significant differences in volume between segmentation procedures (manual–semi‐automatic; Δ_anterior_ = +2.2%; Δ_posterior_ = +3.3%; both p_paired,t_ > 0.1) and they demonstrated high dice overlap (dice_anterior_ = 0.93 ± 0.02; dice_posterior_ = 0.90 ± 0.04), and correlation (R_anterior_^2^ = 93%, p_anterior_ < 10^−10^; R_posterior_^2^ = 10%, p_posterior_ = 0.01). As expected, posterior signal intensity was greater than anterior signal intensity (+101 ± 25% on average, t‐score = 29.4 ± 8.1).

### 3.2 Non‐linear pituitary dynamics before, during, and after pregnancy

**FIGURE 2:**
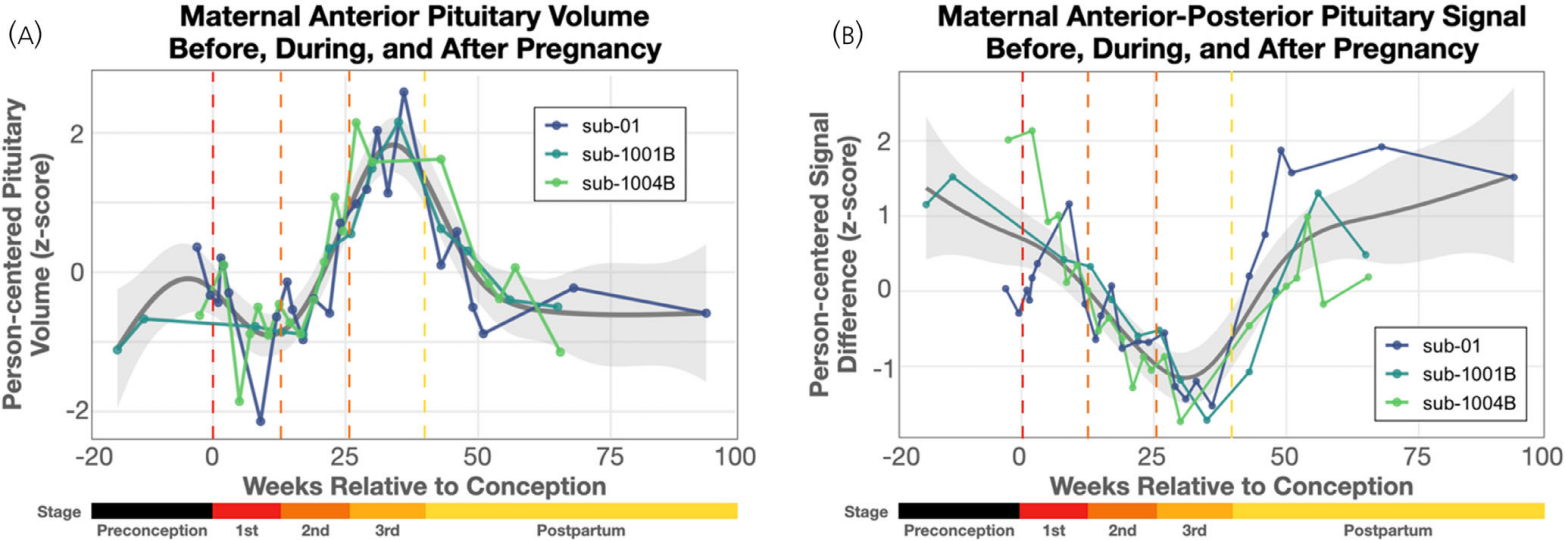
Densely sampled longitudinal dynamics of pituitary volume and signal across pregnancy. (A) Person‐centered anterior pituitary volume changed non‐linearly across gestation, with individual trajectories showing consistent minima near the end of the first trimester and maxima near delivery. (B) Relative T1‐weighted signal intensity differences between anterior and posterior pituitary decreased through pregnancy followed by an abrupt postpartum return to baseline. The dashed vertical lines represent the boundaries delineating preconception, 1st, 2nd, and 3rd trimester, as well as postpartum.

The anterior pituitary lobe followed a consistent nonlinear trajectory across the observation period (Figure 2A). Gestational age explained 73% of adjusted variance in anterior pituitary volume (edf = 7.59, F = 20.2, p_bonf_ < 10^−10^; residual correlation structure ρ = 0.34; AIC = 126.1). Conversely, LOO prediction explained a mean 44% (range 12%–62%) of out‐of‐sample within‐person variance in anterior pituitary volume. Model diagnostics indicated that the smooth term for gestational week was appropriately specified, with no evidence that the model was underfit (k’ = 9, k‐index = 1.13, *p* = 0.77). The non‐linear trajectory of anterior volume can be qualitatively summarized as a modest decrease beginning around the time of conception, reaching a minimum near the end of the first trimester, followed by a strong, roughly linear, increase up to a maximum value near delivery, and followed by a return to baseline after delivery. Quantitatively, based on the first derivative of the model fit, we observed a local minima of −0.9 standardized units at 10.6 gestational weeks, and a local maxima of +1.8 standardized units (or +17.5% by raw volume) at 34.1 weeks gestation. Linear regression analyses during early (−5 to 12 weeks) and late (12 to 40 weeks) windows were conducted for additional insight. We observed linearly decreasing/increasing volumes in the early/late windows (β_early_ = −0.09 SD/week, p_early_ = 0.036; β_late_ = 0.14 SD/week, p_late_ < 10^−10^). No significant volumetric changes in the posterior lobe were observed across the observation period (p_non‐linear_ = 0.79).

Relative signal intensity differences (standardized t‐scores) between anterior and posterior pituitary were observed to be highly nonlinear (Figure 2B). Gestational age explained 66% of adjusted variance in relative signal intensity differences (edf = 5.7, F = 13.4, p_bonf_ < 10^−10^; residual correlation structure ρ = 0.56; AIC = 130.5). Conversely, LOO prediction explained a mean 49% (range 40–58%) of out‐of‐sample within‐person variance in signal intensity differences. Model diagnostics indicated that the smooth term for gestational week was appropriately specified, with no evidence that the model was underfit (k’ = 9, k‐index = 1.03, *p* = 0.53). Qualitatively, this can be described as a roughly linear decrease from near conception to near delivery followed by a rapid return to baseline. Linear regression analyses during the pregnancy window (−5 to 40 weeks) revealed a strong decrease in the difference in signal intensity between anterior and posterior pituitary (β = −0.08 SD/week, p_early_ < 10^−10^).

## 4 DISCUSSION

This study provides the most temporally detailed account to date of pituitary volume dynamics across pregnancy, revealing a rapid, nonlinear pattern of anterior pituitary hypertrophy that peaks in the third trimester and normalizes by approximately 3 months postpartum. The estimated ~17.5% increase in anterior pituitary volume is notably more modest than early claims of a twofold enlargement^11^, ^12^ and aligns more closely with recent work using modern imaging methods suggesting smaller but robust volumetric increases (7–16%).^14^, ^15^ Crucially, the present fine‐grained intraindividual analysis avoids the interpretive pitfalls of cross‐sectional comparisons and provides the highest precision in‐vivo estimate of pregnancy‐related pituitary volume change available.

These findings offer novel insights not only into the magnitude but also the temporal precision of these anterior pituitary‐specific changes during pregnancy. By leveraging dense intraindividual sampling, we were able to identify distinct phases of anterior pituitary expansion: a small decline early in gestation followed by a sustained increase peaking in late gestation, and a rapid return to baseline postpartum. While we observed an early dip in anterior pituitary volume around 10.6 weeks gestation not reported previously, the biological significance of this inflection point remains uncertain, as it has not been reported previously and prospective designs suited to investigate this are lacking. Moreover, it should be noted that this dip was only observed for two of the three women. Although speculative, this observation may reflect a transitional “ramp‐up” period in early pregnancy, when maternal endocrine systems begin to rapidly mobilize in preparation for sustained, intensive hormone production. Alternatively, it is plausible that the observed dip may simply reflect study‐specific methodological factors, such as IVF, the absence of precise delivery age data, and/or sparser first‐trimester sampling in one participant, which limits our ability to fully contextualize relative changes seen across pregnancy. Thus, these results should be interpreted with caution. Future studies with denser early gestational sampling and information about delivery timing data are needed to clarify whether this early inflection point reflects meaningful biological variation or methodological idiosyncrasies.

Another key finding here was the pronounced longitudinal increase in anterior pituitary volume across the second and third trimesters, which was observed in all participants. We report an average increase of ~17.5% in anterior volume, peaking at delivery, that is temporally consistent with pregnancy induced lactotroph hyperplasia and hypertrophy in response to escalating prolactin demand, estrogen exposure, and placental regulatory signals.^20^, ^21^ Importantly, such expansion supports the increases in maternal endocrine capacity necessary for fetal growth, metabolic regulation, and lactation. This physiological basis for the observed changes in volume is further supported by the fact that these processes are largely anterior lobe specific, as observed here. In addition, the observed changes in anterior–posterior signal intensity differences may be similarly indicative of changes in endocrine capacity provided by adaptations in the pituitary during pregnancy. Specifically, while we hypothesize that the massive increases in estrogen, progesterone, and prolactin during pregnancy may be indexed by signal intensity differences, further research incorporating hormone concentrations alongside imaging measures would likely offer physiologically relevant information. In addition, future work would benefit from more signal‐specific MR sequences (e.g., quantitative relaxometry, MR spectroscopy) to better resolve the underlying tissue changes driving these signal shifts.

A primary strength of the current study was the use of a dense sampling design, which offers several key advantages over traditional cross‐sectional or sparse longitudinal designs. First, pregnancy is a time‐limited and nonlinear physiological process; dense sampling allows for the characterization of intraindividual trajectories in a fundamentally novel way that would otherwise be obscured by between‐subjects variability. Second, the richness of this dataset by way of frequent and repeated measurements across the preconception, gestational, and postpartum periods permits fine‐grained modeling of inflection points, nonlinearity, and recovery processes that are often missed in group‐level comparisons. Third, dense intraindividual designs are increasingly recognized as an essential complement to large‐sample studies, particularly when investigating processes that are rapid, variable, and tightly coupled to individual physiology (e.g., hormonal flux, structural plasticity). Importantly, our approach aligns with recent methodological advances in precision neuroscience and endocrinology that prioritize within‐subject modeling of time‐sensitive biological transitions like pregnancy,^2^, ^22^, ^23^ which is vastly consequential for maternal and fetal outcomes.

Despite these strengths, several study limitations warrant acknowledgment. Our sample included only three participants. Although dense sampling provides sufficient power to support the interpretations made here, the small sample size limits generalizability. However, this intensive longitudinal design, focused on within‐subject change rather than between‐subject variability, was deliberately chosen to maximize temporal resolution and mechanistic insight. While the consistency of effect sizes with those reported in larger studies^15^ reinforces alignment with the broader literature, we caution overinterpretation of the current results toward broader generalizability. Moreover, we did not directly measure circulating hormone concentrations, mood, or behavioral outcomes into the present analyses, which will be an essential step in this line of inquiry in order to build more mechanistic models. Additionally, susceptibility artifacts may have impacted volume estimates, though we minimized this risk through quality control, manual segmentation, and within‐subject co‐registration pipelines. Future research integrating multimodal hormone data, expanded imaging around delivery, and whole‐brain analysis will be essential for contextualizing pituitary change as both a biomarker and a mechanism of peripartum neuroplasticity and even fetal programming.

## 5 CONCLUSION

This study offers a high‐resolution view of pituitary plasticity across the human peripartum period, revealing a reproducible, nonlinear pattern of anterior hypertrophy that peaks in late gestation and normalizes postpartum. By combining dense intraindividual sampling with validated segmentation methods, we provide one of the most precise in‐vivo estimates of pregnancy‐related pituitary volume change to date. Critically, these data are openly available and offer a flexible foundation for future large‐scale analyses aimed at linking pituitary dynamics to hormonal, behavioral, and developmental outcomes. Collectively, this framework sets the stage for deeper investigations into peripartum neuroendocrine interactions and their relevance to maternal and infant health.

## AUTHOR CONTRIBUTIONS

Giorgia Picci and Jerod M. Rasmussen contributed to the conceptualization of this project; Risha Arora designed and conducted pre‐processing analyses; Laura Pritschet, Hannah Grotzinger, Kaya Jordan, Emily G. Jacobs, and Elizabeth R. Chrastil designed and collected the densely sampled brain imaging; and all co‐authors contributed to the writing process.

## FUNDING INFORMATION

NICHD R00 HD‐100593 to Jerod M. Rasmussen; NIMH R01 MH‐138481 to Jerod M. Rasmussen; NIGMS P20 GM‐144641 to Giorgia Picci; Ann S. Bowers Women's Brain Health Initiative (Emily G. Jacobs); the University of California Irvine (Elizabeth R. Chrastil); Reprogrants.

## CONFLICT OF INTEREST STATEMENT

All authors have no conflicts of interest to declare.

## Supporting information


**Supplementary Figure S1.** Timing and Density of Observations by Participant. Each participant is represented by a row (e.g., sub‐1004B), with dots (green, teal, purple) representing each observation for by participant before, during, and after pregnancy.
**Supplementary Table S1.** Scaled and Raw Measures of Anterior Pituitary Volume. Model estimates using scaled (left) and raw (right) measures of anterior pituitary volume are shown. Model estimates are across 59 total observations in 3 separate pregnancies.
**Supplementary Figure S2.** Scaled and Raw Measures of Anterior Pituitary Volume. Model estimates using scaled (left) and raw (right) measures of anterior pituitary volume are shown. Model estimates are across 59 total observations in 3 separate pregnancies.
**Supplementary Table S2.** Scaled and Raw Measures of Signal Differences Between Anterior and Pituitary. Model estimates using scaled (left) and raw (right) measures of pituitary signal differences are shown. Model estimates are across 59 total observations in 3 separate pregnancies.
**Supplementary Figure S3.** Scaled and Raw Measures of Signal Differences Between Anterior and Posterior Pituitary. Model estimates using scaled (left) and raw (right) measures of pituitary signal differences are shown. Model estimates are across 59 total observations in 3 separate pregnancies.

## Data Availability

The data that support the findings of this study are available from the corresponding author upon reasonable request.
