# Supplementary material for: Dense sampling for mapping pituitary growth dynamics before, during, and after pregnancy
Source: J Neuroendocrinol. 2026 Feb 5;38(2):e70141. doi: 10.1111/jne.70141 (PMC12875737; doi:10.1111/jne.70141)
Supplement: Supplementary file 1 — Supplementary Figure S1. Timing and Density of Observations by Participant. Each participant is represented by a row (e.g., sub‐1004B), with dots (green, teal, purple) representing each observation for by participant before, during, and after pregnancy. Supplementary Table S1. Scaled and Raw Measures of Anterior Pituitary Volume. Model estimates using scaled (left) and raw (right) measures of anterior pituitary volume are shown. Model estimates are across 59 total observations in 3 separate pregnancies. Supplementary Figure S2. Scaled and Raw Measures of Anterior Pituitary Volume. Model estimates using scaled (left) and raw (right) measures of anterior pituitary volume are shown. Model estimates are across 59 total observations in 3 separate pregnancies. Supplementary Table S2. Scaled and Raw Measures of Signal Differences Between Anterior and Pituitary. Model estimates using scaled (left) and raw (right) measures of pituitary signal differences are shown. Model estimates are across 59 total observations in 3 separate pregnancies. Supplementary Figure S3. Scaled and Raw Measures of Signal Differences Between Anterior and Posterior Pituitary. Model estimates using scaled (left) and raw (right) measures of pituitary signal differences are shown. Model estimates are across 59 total observations in 3 separate pregnancies. [file JNE-38-e70141-s001.docx]

**Supplementary Materials**


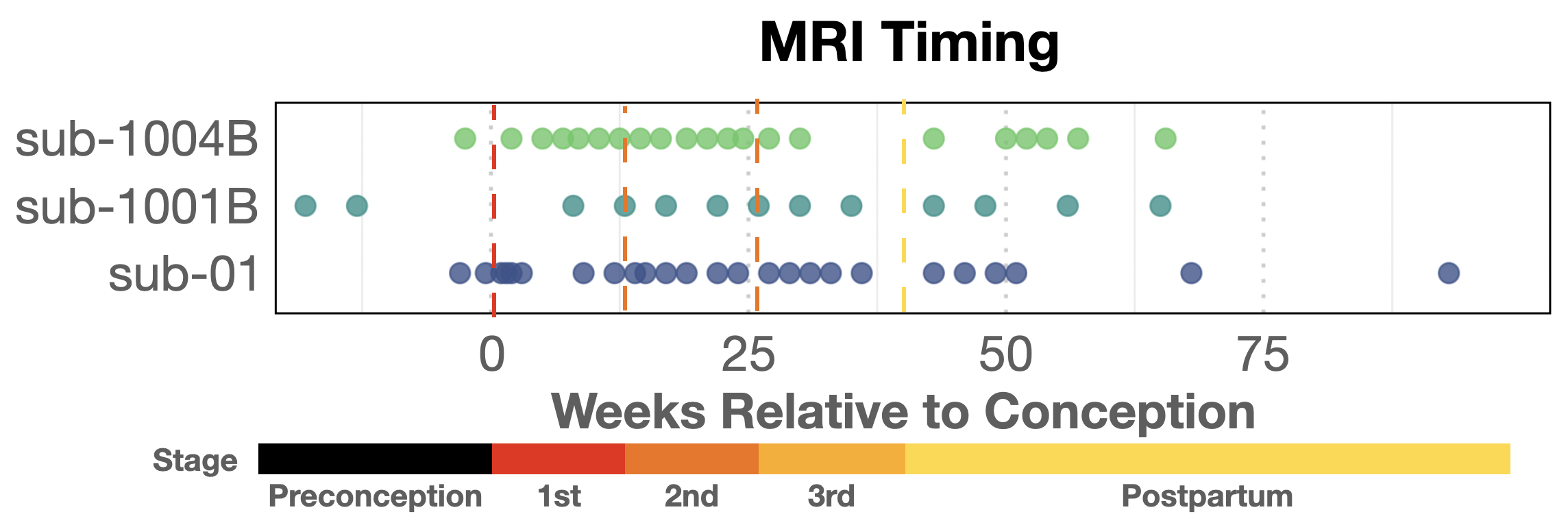


**Supplementary Figure S1. Timing and Density of Observations by Participant.** Each participant is represented by a row (e.g., sub-1004B), with dots (green, teal, purple) representing each observation for by participant before, during, and after pregnancy.

***GAMM Models Using Raw Measures***

*Raw Measure Models*

Models used in the main manuscript were re-ran using raw (non-scaled) measures of anterior pituitary volume and anterior/posterior signal differences. Here, we report full models specified for both scaled and non-scaled measures, along with their GAMM fits. Largely, we observed qualitatively similar trajectories with increased uncertainty in the estimate.

| **Dependent** | **Anterior Pit. Volume (z)** | **Anterior Pit. Volume (raw)** |
| --- | --- | --- |
| **Intercept (SE)** | 0.0 (0.1) | 700.2 (91.0) |
| **EDF** | 7.6 | 7.1 |
| **F** | 20.2 | 12.3 |
| **p** | <10E-10 | <10E-10 |
| **Rho** | 0.34 | 0.72 |
| **AIC** | 126.1 | 567.2 |

**Supplementary Table S1. Scaled and Raw Measures of Anterior Pituitary Volume.** Model estimates using scaled (left) and raw (right) measures of anterior pituitary volume are shown. Model estimates are across 59 total observations in 3 separate pregnancies.


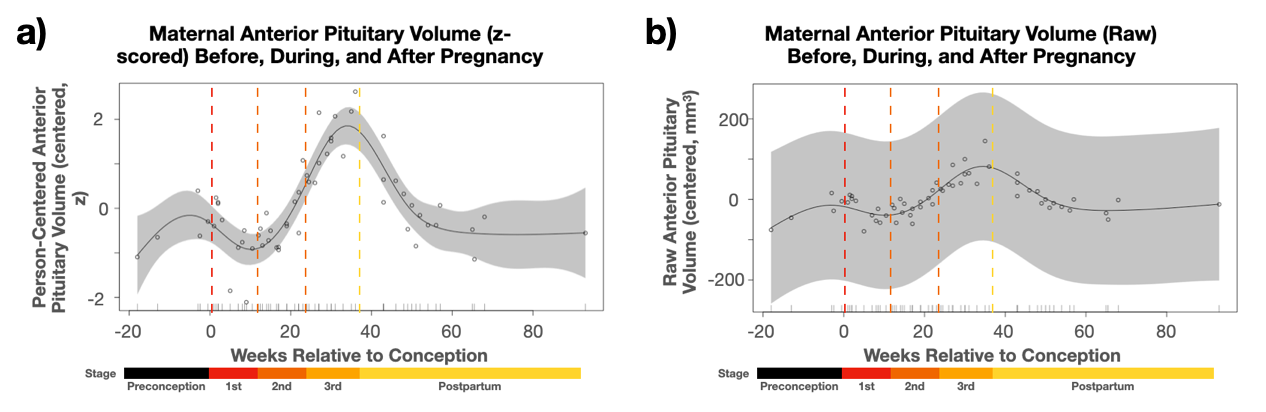


**Supplementary Figure S2. Scaled and Raw Measures of Anterior Pituitary Volume.** Model estimates using scaled (left) and raw (right) measures of anterior pituitary volume are shown. Model estimates are across 59 total observations in 3 separate pregnancies.

| **Dependent** | **Signal Intensity Diff. (z)** | **Signal Intensity Diff. (raw)** |
| --- | --- | --- |
| **Intercept (SE)** | 0.0 (0.1) | 29.6(5.0) |
| **EDF** | 5.7 | 5.4 |
| **F** | 13.4 | 11.1 |
| **p** | <10E-10 | <10E-10 |
| **Rho** | 0.56 | 0.63 |
| **AIC** | 130.5 | 273.5 |

**Supplementary Table S2. Scaled and Raw Measures of Signal Differences Between Anterior and Pituitary.** Model estimates using scaled (left) and raw (right) measures of pituitary signal differences are shown. Model estimates are across 59 total observations in 3 separate pregnancies.


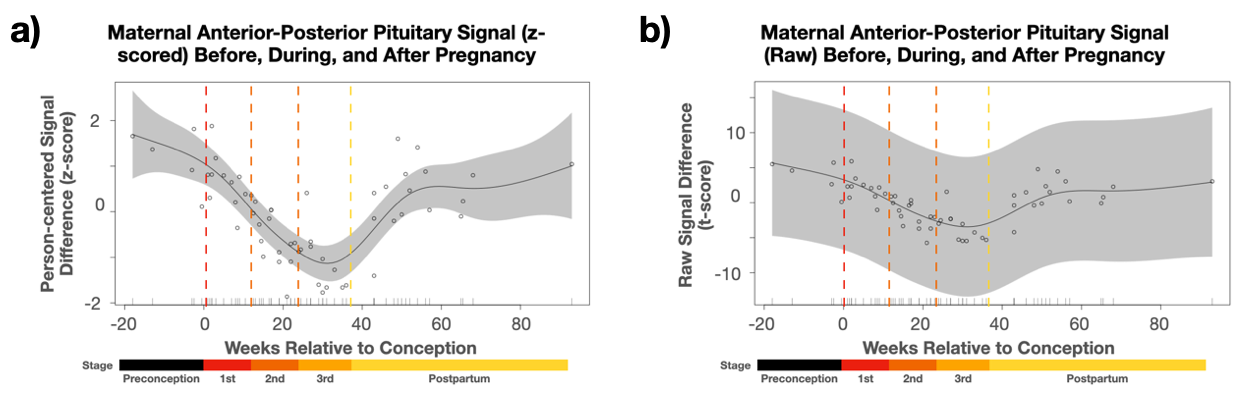


**Supplementary Figure S2. Scaled and Raw Measures of Signal Differences Between Anterior and Pituitary.** Model estimates using scaled (left) and raw (right) measures of pituitary signal differences are shown. Model estimates are across 59 total observations in 3 separate pregnancies.
